# Global gridded crop harvested area, production, yield, and monthly physical area data circa 2015

**DOI:** 10.1038/s41597-021-01115-2

**Published:** 2022-01-20

**Authors:** Danielle Grogan, Steve Frolking, Dominik Wisser, Alex Prusevich, Stanley Glidden

**Affiliations:** 1 Institute for the Study of Earth, Oceans, and Space, University of New Hampshire, Durham, USA; 2 Food and Agricultural Organization of the United Nations, Rome, Italy

**Keywords:** Environmental impact, Agroecology, Hydrology, Climate sciences, Biogeochemistry

## Abstract

Here we provide an update to global gridded annual and monthly crop datasets. This new dataset uses the crop categories established by the Global Agro-Ecological Zones (GAEZ) Version 3 model, which is based on the Food and Agricultural Organization of the United Nations (FAO) crop production data. We used publicly available data from the FAOSTAT database as well as GAEZ Version 4 global gridded dataset to generate circa 2015 annual crop harvested area, production, and yields by crop production system (irrigated and rainfed) for 26 crops and crop categories globally at 5-minute resolution. We additionally used available data on crop rotations, cropping intensity, and planting and harvest dates to generate monthly gridded cropland data for physical areas for the 26 crops by production system. These data are in standard georeferenced gridded format, and can be used by any global hydrology, land surface, or other earth system model that requires gridded annual or monthly crop data inputs.

## Abstract (summary)

**Table:** 

Measurement(s)	crop yield • crop harvest area • crop production • area of cropland
Technology Type(s)	national reporting
Sample Characteristic - Environment	agriculture
Sample Characteristic - Location	global

Machine-accessible metadata file describing the reported data: 10.6084/m9.figshare.16924087

## Background & Summary

Cropland covers about 10% of the Earth’s land surface, making it an essential component of all land surface and Earth system models. Croplands also produce feed, food, fiber, and fuel, and are the dominant consumer of freshwater globally, making them an important component of global hydrologic, economic, and multi-sector dynamics models. While there are currently available datasets that provide annual cropland extent (e.g., the History Database of the Global Environment (HYDE 3.2)^1^, or the SEDAC Croplands v1^2,3^), there is a need for information beyond the scope of those datasets. First, cropland extent alone cannot provide information on the production (kg) or yield (production per area) of a crop, which is important for understanding biogeochemical cycling as well as food production. Further, to fully evaluate the impact of crops on water use, evaporative fluxes, and energy fluxes, there is need for a dataset that provides the monthly temporal resolution of cropping activity, along with disaggregation by crop type and irrigated versus rainfed management. The MIRCA2000^4^ dataset provides these monthly and crop-wise resolution data, but only circa year 2000. Globally, cropland area increased by approximately 5% from 2000 to 2015 (~775,000 km^2^)^5^, though with great spatial variation even in the direction of this change (positive vs negative) at the country, watershed, and grid cell level^1,5^.

Here, we address these cropland data needs by producing a year 2015 gridded annual harvested area, production, and yield dataset for 26 crops and crop categories (e.g., wheat, vegetables), disaggregated by irrigated and rainfed management. We then generate a monthly gridded cropland dataset for each of those crops, indicating which months the crop is actually grown in the field, such that the monthly area is consistent with the annual harvested area for each crop. The foundation for this year 2015 cropland data is a gridded cropland dataset circa year 2010 generated for use by the Global Agro-Ecological Zones model v4 (GAEZ 2010)^6,7^. The GAEZ v4 methodology^6^ couples national aggregate statistical data on crops and irrigation from FAO, with detailed spatial data, taken from a variety of published sources, on cropland distribution, irrigation distribution, soil properties, climate, human population (to allocate land area to built infrastructure), livestock population (to allocate land area to livestock), protected lands, lakes and wetlands, observed crop phenology and crop calendars, as well as available sub-national crop statistics. The ancillary spatial data are used to generate a series of gridded intermediate products, e.g., average attainable irrigated and rainfed yields, irrigated and rainfed multi-cropping intensity class. These intermediate spatial products are used to downscale the national aggregate statistical crop data from FAOSTAT^5^ (average of period 2009–2011) to 5-minute gridded products of irrigated and rainfed harvest area, production, and yield, as well as value of crop production. The downscaling process is a sequential, iterative rebalancing procedure that relies on optimization principles^8^ suitable for situations when the aggregate observed information is available (here FAO national crop statistics), constrained by the priors (i.e., the intermediate spatial maps), other available statistical data, and expert opinion^6^. Here we present our methodology for updating the GAEZ 2010 products to 2015, providing more recent data on production, yield, and harvested areas; these data are available to the wider global modeling community. The new dataset is referred to here as GAEZ+ 2015.

Updating to 2015 was done using FAOSTAT^5^ country-level information on crop harvest area, crop production, and livestock herd size changes from 2010 to 2015, along with the FAO GeoNetwork’s Global Administrative Unit Layer (GAUL)^7^ to map 5 minute grid cells to countries. We used the MIRCA2000^4^ information on multi-cropping patterns and monthly crop calendars to convert the updated GAEZ+ 2015 crop harvest areas into monthly cropland physical areas by crop and production system. This method uses national-level data to update a gridded product; as such, it is important to note the limits of this data set. GAEZ+ 2015 is not based on remote sensing observations of cropland presence or absence in a given grid cell, and therefore represents aggregate changes in cropland extent but not high-resolution changes. Further, information on crop calendars and rotations is taken from the MIRCA2000^4^ dataset, so GAEZ+ 2015 does not include information on changes to crop calendars or rotations in the past 15 years. This data product is meant to be an intermediate data set for use by those who need crop layers consistent with country level FAO-reported 2015 statistics while we await the release of other products such as the crop- and production-specific datasets that are promised from the Global Food Security-Support Analysis Data (GFSAD)^9^ and MapSPAM^10^, both of which use remote sensing to better identify changes in crop locations and calendars.

The data provided here follow commonly used file formats (geotiff and netCDF) and metadata standards familiar to global gridded modeling communities. The re-use value is high, as there are a number of Global Hydrologic Models and Land Surface Models that use this type of data. Additionally, interdisciplinary research teams are increasingly using global gridded crop data, e.g., in global economic models such as SIMPLE-G^11^ and integrated assessment models such as the Global Change Analysis Model GCAM^12^. Further, this dataset uses the GAEZ crop list and ensures consistency with FAO administrative level agricultural data, thereby connecting this dataset to already widely used crop categories and more aggregate publicly available data that is already in use. FAO is currently using the GAEZ+ 2015 data in an ongoing study.

## Methods

**Fig. 1:**
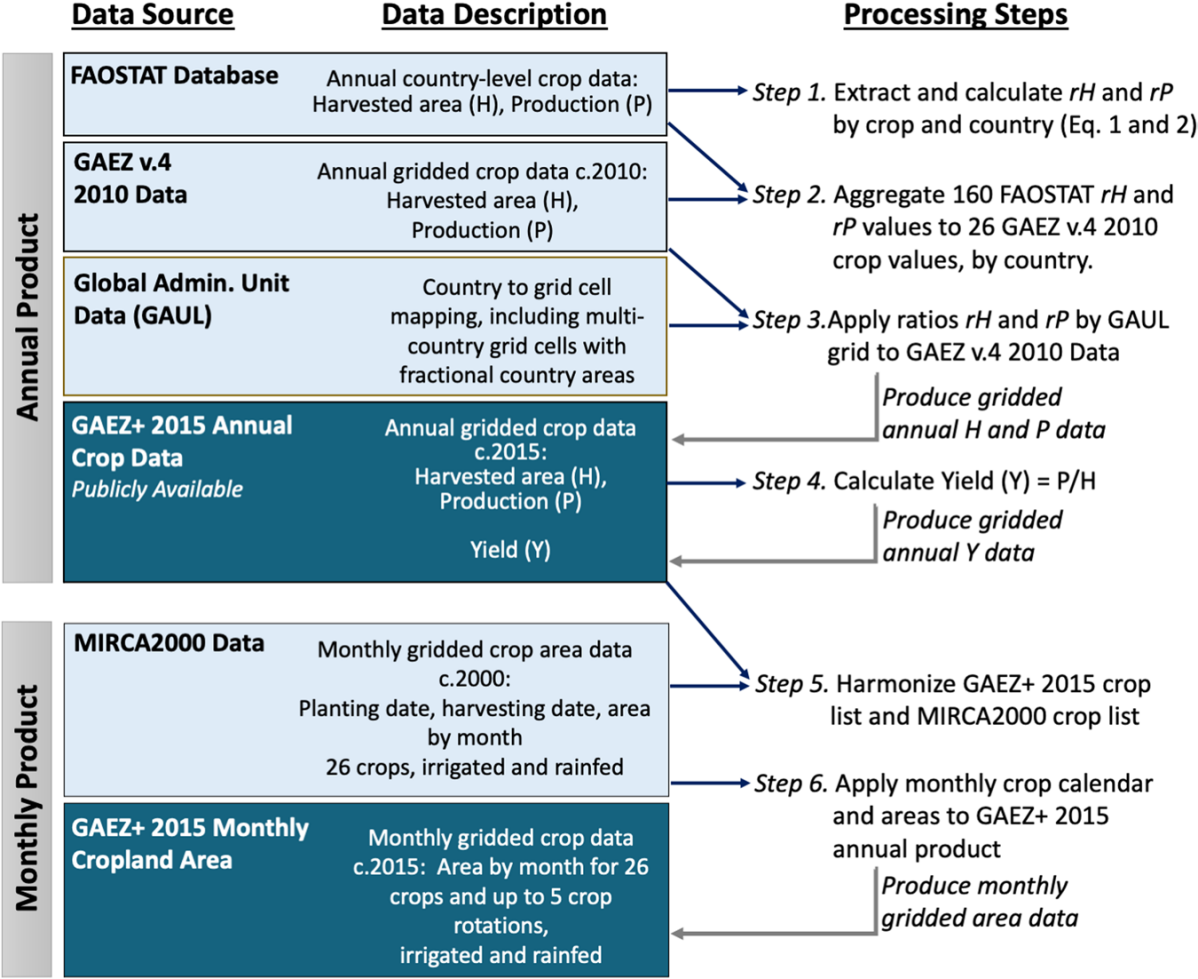
Schematic overview of annual and monthly data production methods. The GAEZ+ 2015 products described in this paper are in dark blue boxes; publicly available data used are in light blue. Dark blue arrows indicate which data are used in each processing step, and grey arrows from steps to data show which steps result in final GAEZ+ 2015 data products. The processing steps listed here are referred to in the Methods section text.

Here we describe methods for the GAEZ+ 2015 Annual Crop Data, and the GAEZ+ 2015 Monthly Cropland Data. The Annual Crop Data was generated first, then the Monthly Cropland Data was calculated based on the Harvest Area results of the Annual Data (Fig. 1).

### GAEZ+ 2015 Annual Crop Data Methods

The GEAZ+ 2015 Annual Crop Data updates the 2010 GAEZ v4 crop harvest area, yield, and production maps^6,7^ (identified as Theme 5 in ref. ^7^) using national-scale data on the change in crop harvested area and livestock numbers from 2010 to 2015, based on statistics for 160 crop groups, and cattle and buffalo, from FAOSTAT^5^.

Three datasets were used to produce GAEZ+ 2015 Annual Crop Data:

1. FAOSTAT crop production domain: annual, country-level data on crop harvested area (*H*) and crop production (*P*) for each crop from the FAOSTAT database (Table 1)

**Table 1:** GAEZ and FAOSTAT crop harmonization.

GAEZ Crop Name	FAOSTAT Crop Name (production domain crop_code)
Wheat	Wheat (15)
Rice	Rice_paddy (27)
Maize	Maize (56)
Sorghum	Sorghum (83)
Millet	Millet (79)
Barley	Barley (44)
Other_cereals	Rye (71), Oats (75), Buckwheat (89), Quinoa (92), Fonio (94), Triticale (97), Canary_seed (101), Grain_mixed (103), Cereals_nes (108)
Potato_&_Sweet_potato	Potatoes (116), Sweet_potatoes (122)
Cassava	Cassava (125)
Yams_and_other_roots	Yautia (cocoyam) (135), Taro (cocoyam) (136), Yams (137), Roots_and_tubers_nes (149)
Sugarbeet	Sugar_beet (157)
Sugarcane	Sugar_cane (156)
Pulses	Beans_dry (176), Broad_beans_dry (181), Peas_dry (187), Chick_peas (191), Cow_peas_dry (195), Pigeon_peas (197), Lentils (201), Bambara_beans (203), Pulses_nes (211)
Soybean	Soybeans (236)
Rapeseed	Rapeseed (270)
Sunflower	Sunflower_seed (267)
Groundnut	Groundnuts_with_shell (242)
Oil_palm_fruit	Oil_Palm_Fruit (254)
Olives	Olives (260)
Cotton	Seed_Cotton (328)
Tobacco	Tobacco_unmanufactured (826)
Banana	Bananas (486), Plantains (489)
Stimulants	Coffee_green (656), Cocoa_beans (661), Tea (667), Maté (671)
Vegetables	Cabbages (358), Artichokes (366), Asparagus (367), Lettuce_and_chicory (372), Spinach (373), Cassave_Leaves (378), Tomatoes (388), Cauliflowers_and_broccoli (393), Pumpkins_squash_gourds (394), Cucumbers_and_gherkins (397), Eggplants (aubergines) (399), Chillies_peppers_green (401), Onions_shallots_green (402), Onions_dry (403), Garlic (406), Leeks (407), Beans_green (414), Peas_green (417), Vegetables_legum._nes (420), String_beans (423), Carrots_and_turnips (426), Okra (430), Maize_green (446), Mushrooms_and_truffles (449), Chicory_roots (459), Carobs (461), Vegetables_fresh_nes (463), Chillies_and_peppers_dry (689)
Crops_NES	Brazil_nuts_with_shell (216), Cashew_nuts_with_shell (217), Chestnut (220), Almonds_with_shell (221), Walnuts_with_shell (222), Pistachios (223), Kola_nuts (224), Hazelnuts_with_shell (225), Areca_nuts (226), Nuts_nes (234), Coconuts (249), Karite_nuts (sheanuts) (263), Tung_nuts (275), Melonseed (299), Kapok_Fruit (310), Oranges (490), Tangerines (495), Lemons_and_limes (497), Grapefruit_(incl._pomelos) (507), Fruit_citrus_nes (512), Apples (515), Pears (521), Quinces (523), Apricots (526), Cherries_sour (530), Cherries (531), Peaches_and_nectarines (534), Plums_and_sloes (536), Fruit_stone_nes (541), Fruit_pome_nes (542), Strawberries (544), Raspberries (547), Gooseberries (549), Currants (550), Blueberries (552), Cranberries (554), Berries_nes (558), Grapes (560), Watermelons (567), Melons_other (568), Figs (569), Mangoes_guavas (571), Avocados (572), Pineapples (574), Dates (577), Persimmons (587), Cashewapple (591), Kiwi_fruit (592), Papayas (600), Fruit_tropical_fresh_nes (603), Fruit_fresh_nes (619), Sugar_crops_nes (161), Vetches (205), Lupins (210), Castor_oil_seed (265), Jojoba_Seeds (277), Safflower_seed (280), Sesame_seed (289), Mustard_seed (292), Poppy_seed (296), Tallowtree_Seeds (305), Linseed (333), Hempseed (336), Oilseeds_nes (339), Hops (677), Pepper (piper_spp.) (687), Vanilla (692), Cinnamon (canella) (693), Cloves (698), Nutmeg_mace_cardamoms (702), Anise_badian_fennel (711), Ginger (720), Spices_nes (723), Peppermint (748), Pyrethrum_dried (754), Flax_fibre_and_tow (773), Hemp_tow_waste (777), Jute (780), Bastfibres_other (782), Ramie (788), Sisal (789), Agave_fibres_nes (800), Manila_fibre_(abaca) (809), Fibre_crops_nes (821)
Fodder_crop*	*Cabbages_for_fodder (644), Pumpkins_for_fodder (645), Turnips_for_fodder (646), Beets_for_fodder (647), Carrots_for_fodder (648), Swedes_for_fodder (649), Vegetables_&_roots_fodder (655), Forage_&_Silage_crops (n.a.), Forage_products (651), Forage_&_silage,_maize (636), Forage_&_silage,_sorghum (637), Forage_&_silage,_rye_grass (638), Forage_&_silage,_grasses_nes (639), Forage_&_silage,_clover (640), Forage_&_silage,_alfalfa (641), Forage_&_silage,_green_oilsd (642), Forage_&_silage,_legumes (643)*

*The FAOSTAT fodder and silage crops no longer have harvested area and production reported in FAOSTAT.
2. GAEZ v4^6,7^ gridded global annual harvested area, yield, and production by crop for the 26 FAOSTAT crops and crop categories at 5-minute resolution
3. Global Administrative Unit Layer (GAUL 2012)^13^ data. GAUL 2012 reports the fraction of each global 5-minute grid cell that falls within a given country or disputed territory. There are 275 unique global administrative units.

*Step 1. Calculate crop changes from 2010 to 2015 by country:*

For each country, we extracted the harvested area (*H*) and crop production (*P*) for each of the 160 FAOSTAT crop categories, *c*, from the FAOSTAT database. We averaged three years (2009–2011) of annual national crop harvested area data to represent 2010 national crop harvest area, *H*_2010_, and three years (2014–2016) of annual crop harvested area data to represent 2015 national crop harvest area, *H*_2015_, then calculated a ratio, *rH*_*c*,_ of 2015 to 2010 harvested areas for each crop *c* in each country, and equivalently, for crop production:

1 $$r{H}_{c}={H}_{2015}/{H}_{2010}$$

2 $$r{P}_{c}={P}_{2015}/{P}_{2010}$$

This results in 160 *rH* and *rP* values per country. If harvest area and production values for a particular crop are zero or unreported in the FAOSTAT data, then *rH*_*c*_ and *rP*_*c*_ are both set to 1.0 (i.e., no change from 2010 to 2015). Three years of data are averaged (2009 – 2011 and 2014 – 2016) to account for missing data for some country/year combinations and to avoid emphasizing reported outliers.

*Step 2. Aggregate FAOSTAT-based ratios to the GAEZ crop categories:*

We followed the crop aggregation methods of the GAEZ model to aggregate the FAOSTAT crop list (160 unique crops as of 2019) to 26 crops (see Table 1). For each of the 26 GAEZ crop categories, if there is more than one matching FAOSTAT crop (see Table 1) then we applied an area-weighted average (based on FAOSTAT year 2015 harvested area) of the FAOSTAT crops within each country to the *rH* and *rP* values for that crop and country. This results in 26 *rH* and *rP* values per country. There was one exception to this: the GAEZ_2010 crop category ‘fodder crops’ was an aggregate of 17 FAOSTAT crops (see Table 1) for which harvest area data are no longer reported on FAOSTAT; i.e., GAEZ_2010 had obtained FAOSTAT data on fodder crops circa 2010, but FAOSTAT no longer provides any data on fodder crops for any year. We assumed that the 2010 to 2015 fractional change in fodder crop harvest area in each country was proportional to the change in the FAOSTAT reported national herd sizes for cattle and buffalo livestock data^5^ for that country, following the same methodology as for crop harvested area change (see Step 2 below). This method assumes a negligible international trade of fodder crops as indicated by bilateral trade matrices available from FAOSTAT.

*Step 3. Apply country-level ratios to grid cells:*

Calculated country-level ratios were then applied to each grid cell *k*, using the GAUL_2012^13^ definitions for which grid cells fall within which countries. Some grid cells are split between two or more countries. In this case, all model output variables for the grid cell are divided between the countries based on the fraction of grid cell area falling within the country *i*:

3 $${H}_{c,2015}^{k}={H}_{c,2010}^{k}{\sum }_{i}\,{f}_{i}^{k}r{H}_{c,i}$$

4 $${P}_{c,2015}^{k}={P}_{c,2010}^{k}{\sum }_{i}\,{f}_{i}^{k}r{P}_{c,i}$$

where ${H}_{c,2015}^{k}$ is the year 2015 harvested area (or production) for crop *c* in grid cell *k*; ${f}_{i}^{k}$ is the fraction of country *i* in grid cell *k*, and *rH*_*c,i*_ and *rP*_*c,i*_ are the ratios for crop *c* in country *i* as calculated in Eqs. 1 and 2. This results in 26 *H* and *P* values per grid cell. If the sum of all crop harvest areas exceeds 99% of the grid cell area, all crop harvest areas are reduced equally to fit within 99% of the area.

*Special Case: Sudan*

FAOSTAT data for years before 2011 report data for Sudan, and for South Sudan and Sudan after 2011. To compute the ratios for these grid cells, we split the 2010 data for Sudan into a virtual ‘North’ Sudan and ‘South_Sudan’, using the data for the year 2012, which was reported for both countries. We then used these generated 2010 data and applied the same methodology as described above to calculate changes in harvested areas and production in all grid cells in both countries.

*Special Case: Small regions and islands*

Forty-nine countries - generally small regions or islands - had no data reported for crop harvested area by FAOSTAT. We assumed that there was no change in crop harvested area for the grid cells in these countries. Note that many may have had zero ha as previously-reported crop area in GAEZ v4. These countries are (the number following each region is the region’s number in ADM0_CODE in the GAUL_2012 data^13^):

*Anguilla (9), Aruba (14), Ashmore_and_Cartier_Islands (16), Azores_Islands (74578), Baker_Island (22), Bassas_da_India (25), Bird_Island (32), Bouvet_Island (36), British_Indian_Ocean_Territory (38), Christmas_Island (54), Clipperton_Island (55), Cocos (Keeling)_Islands (56), Europa_Island (80), French_Southern_and_Antarctic_Territories (88), Glorioso_Island (96), Greenland (98), Guernsey (104), Heard_Island_and_McDonald_Islands (109), Howland_Island (112), Isle_of_Man (120), Jarvis_Island (127), Jersey (128), Johnston_Atoll (129), Juan_de_Nova_Island (131), Kingman_Reef (134), Kuril_islands (136), Madeira_Islands (151), Mayotte (161), Midway_Island (164), Navassa_Island (174), Netherlands_Antilles (176), Norfolk_Island (184), Northern_Mariana_Islands (185), Palmyra_Atoll (190), Paracel_Islands (193), Pitcairn (197), Saint_Helena (207), Scarborough_Reef (216), Senkaku_Islands (218), South_Georgia_and_the_South_Sandwich_Islands (228), Spratly_Islands (230), Svalbard_and_Jan_Mayen_Islands (234), Tromelin_Island (247), Turks_and_Caicos_Islands (251), United_States_Virgin_Islands (258), Wake_Island (265), Gibraltar (95), Holy_See (110), Liechtenstein (146)*.

*Special Case: Disputed Areas*

Some grid cells in the GAUL_2012^13^ cell-table database are assigned to nine disputed areas, rather than to specific countries. We assumed that there was no change in crop harvested area or production from 2010 to 2015 for grid cells these disputed areas. These areas are (the number following each region is the region’s number of the ADM0_CODE in the GAUL_2012^13^ data):

*Abyei (102), Aksai_Chin (2), Arunachal_Pradesh (15), China/India (52), Hala’ib_Triangle (40760), Ilemi_Triangle (61013), Jammu_and_Kashmir (40781), Ma’tan_al-Sarra (40762), Falkland_Islands_(Malvinas) (81)*.

*Step 4. Compute 2015 crop yields:*

Crop yields were computed for each crop, *c*, and grid cell, *k*, as the ratio of crop production to crop harvest area (if harvest area, *H*_*c,k,2015*_, is zero, then yield, *Y*_*c,k,2015*_, is set to zero):

5 $${Y}_{c,k,2015}={P}_{c,k,2015}/{H}_{c,k,2015}$$

The resulting gridded global data are:

A. GAEZ+ 2015 Crop Harvest Area^14^
B. GAEZ+ 2015 Crop Yield^15^
C. GAEZ+ 2015 Crop Production^16^

This new data product consists of 156 data files in geotiff format, one rainfed harvested area file and one irrigated harvested area file for each crop harvest area (1000 ha (10^7^ m^2^) per 5-minute grid cell), crop production (1000 tonnes (10^6 ^kg) per 5-minute grid cell), and crop yield (tonnes per ha (10^−1 ^kg m^−2^) per 5-minute grid cell), for each of the 26 GAEZ crops or crop categories in Table 1.

### GAEZ+ 2015 monthly cropland area methods

Two datasets were used to produce monthly cropland area by crop and by irrigated vs rainfed management. These are:

1. GAEZ+ 2015 Annual Harvested Area^14^ (as developed above)
2. MIRCA2000 cropland area^4^

*Step 5. Harmonize the GAEZ+ 2015 and MIRCA2000 crop lists*

The MIRCA2000^4^ cropland product provides monthly growing area grids (gridded physical cropland area) for 26 irrigated and rainfed crops and crop categories, as well as cropping calendars that identify the planting month and harvesting month for each crop (via ‘subcrops’ – see below). However, the MIRCA2000 crop list is not the same as the GAEZ+ 2015 crop list; we matched each crop type in the GAEZ+ 2015 crop list to a crop type in the MIRCA2000 crop list to enable the application of MIRCA2000 crop calendars to GAEZ+ 2015 crops (Table 2). Out of the 26 GAEZ+ 2015 crops, 18 had clear 1:1 matching crop categories within MIRCA2000. The remaining 8 crops were matched based on general crop characteristics, i.e., annual vs. perennial, or to unmatched MIRCA2000 cereals.

**Table 2:** List of GAEZ crop categories used in all GAEZ+ 2015 products, as well as the matching between GAEZ+ 2015 crops and MIRCA2000^4^ crop categories for the purposes of producing GAEZ+ 2015 monthly cropland data.

GAEZ crop	MIRCA2000 crop	1:1 Matching	Removal Order
Banana	Others perennial	No	3
Barley	Barley	YES	18
Cassava	Cassava	YES	11
Cotton	Cotton	YES	4
Crops NES	Others perennial AND others annual	No	2
Foddercrops	Fodder grasses	no	2
Groundnut	Groundnut	YES	6
Maize	Maize	YES	20
Millet	Millet	YES	16
Oil palm fruit	Oil palm	YES	8
Olives	Others perennial	No	3
Other cereals	Rye AND Millet	No	17
Potato & Sweet potato	Potatoes	YES	12
Pulses	Pulses	YES	5
Rapeseed	Canola	YES	7
Rice	Rice	YES	19
Sorghum	Sorghum	YES	15
Soybean	Soybeans	YES	14
Stimulants	Others annual	No	1
Sugarbeet	Sugarbeet	YES	9
Sugarcane	Sugarcane	YES	10
Sunflower	Sunflower	YES	13
Tobacco	Others annual	No	1
Vegetables	Others annual	No	1
Wheat	Wheat	YES	21
Yams & other roots	Others annual	No	1

The Removal Order column lists the order in which cropland (rainfed first, then irrigated) are removed from a grid cell in the case that the monthly cropland area exceeds the grid cell area.

An essential component of the MIRCA2000 cropland dataset is the identification of subcrop categories within each crop category to split crops into areas grown in different seasons, or crops with different planting and harvesting dates within the same season. Up to 5 subcrops can be defined to represent such multi-cropping practices. Below, we use the following notation:

*H*_*G*_ = annual harvested area from the GAEZ+ 2015 product for a given crop

*H*_*M* = _annual harvested area calculated from the MIRCA2000 data for a given crop

*A*_*M,n*_ = cropland area of MIRCA2000 crop, subcrop n, by month

*A*_*G,n*_ = cropland area of GAEZ+ 2015 crop, subcrop n, by month

*A*_*G*_ = cropland area of GAEZ+ 2015 crop, by month

*Step 6. Apply MIRCA2000 monthly crop calendars to GAEZ+ 2015 annual data*

To generate the monthly cropland physical area of GAEZ+ 2015 crops, we followed these steps for each GAEZ crop in each grid cell:

1. For a given GAEZ crop in a given grid cell, is the area reported >0 for the matching MIRCA2000 crop?
  - If YES, then use the MIRCA2000 data for the grid cell and crop considered.
  - If NO, then find the closest grid cell with the matching MIRCA2000 crop category, and apply the MIRCA2000 crop rotation from that grid cell to the given crop/grid cell combination for the following steps.
2. Does the matching MIRCA2000 crop category (Table 1) have more than 1 subcrop?
  - If NO, then *A*_*G*_ = *H*_*G*_ for all months of the cropping season, as defined by the MIRCA2000 crop calendar.
  - If YES, then for each subcrop category *n*, apply the ratio of *A*_*M,n*_*/H*_*M*_ to *H*_*G*,_ then sum the subcrop areas within each month such that:
  $${A}_{G}=\sum _{n}\frac{{A}_{M,n}}{{H}_{M}}{H}_{G}$$
3. For each month and each grid cell, check if the sum of all crops (irrigated and rainfed) is greater than the 99% of area of the grid cell. We assume that at least 1% of land must be retained as non-cropland for agricultural infrastructure such as roads, buildings, irrigation infrastructure, and other landcovers (e.g. rivers, wetlands).
  - If NO, then no further processing is done.
  - If YES, then reduce crop area by the excess value based on a removal order (Table 2). Rainfed crops have higher removal order numbers for the excess truncation (starting with 1) before removing irrigated crops, until the cell area is not exceeded. A large removal number (e.g., 20) indicates that the crop’s land is unlikely to be removed. Large priority numbers are given to the staple crops to ensure these important food producing lands are consistent with FAOSTAT country data.

The maximum monthly amount of physical cropland that was removed by step 3 is 711,543 ha, which is 0.05% of total global cropland physical area.

**Fig. 2:**
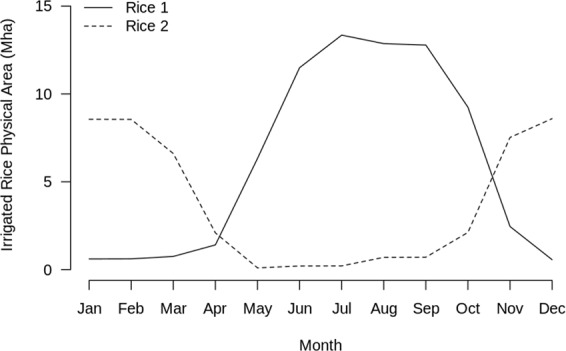
Aggregate monthly cropland physical area for Rice 1 and Rice 2 subcrops from monthly GAEZ+ 2015 over the northern hemisphere shows the two main rice-growing seasons. This seasonality is the result of combining GAEZ+ 2015 annual data with the MIRCA20004 crop calendars and subcrop divisions.

The resulting global gridded data from *Step 6* are monthly time series of cropland physical area by crop, subcrop, and production system, called GAEZ+_2015 Monthly Cropland Data^17^. Combining the MIRCA2000 crop calendar and subcrop rotation information with the GAEZ+ 2015 annual data allows for the representation of crop seasonality; e.g., Fig. 2 shows the aggregate monthly cropland physical area for Rice 1 and Rice 2 (two sub-crops of rice) over the northern hemisphere, clearly illustrating the two main rice-growing seasons.

## Data Records

***GAEZ***+ ***2015 Annual Crop Data***^14–16^:

**File format**: geotiff

**File naming convention:** GAEZAct2015_{*Variable*}_{*Cropname*}*_*{*Management*}.tif

Where {*Variable*} is one of: *HarvArea, Production* or Y*ield*.

{*Cropname*} is one of the names from column 1 in Table 1.

{*Management*} is one of: *Irrigated*, *Rainfed*, *Total* or *Mean*.

**Date Produced**: May 2020.

**Spatial Metadata**:

Extent: X: −180 to +180

Extent Y: −90 to +90 Resolution: 0.083333 decimal degrees (5 arcminutes)

Coordinate reference system: longitude/latitude (WGS84 datum)

Projection in PROJ.4 notation: “+proj = longlat + datum = WGS84”

**Units**:

Crop Harvest Area: 1000 ha (10^7^ m^2^) per 5-arcminute grid cell

Crop Production: 1000 tonnes (10^6 ^kg) per 5-arcminute grid cell

Crop Yield: tonnes per ha (10^−1 ^kg m^−2^) per 5-arcminute grid cell

No data value: Oceans, open-water, and Antarctica in the geotiff files have the no-data value of −3.39999999999999996e + 38.

**Repository**: Harvard dataverse

**GAEZ+ 2015 Monthly Cropland Data** ^17^

**File format**: netCDF v.4 with default internal compression (level 7)

**File naming convention:** GAEZ_CropArea_{*Cropname*}*_{subcrop*}_{*Management*}.nc

Where: {*Cropname*} is one of the names from column 1 in Table 1.

{*subcrop*} is a number (1 through 5) indicating crop rotations.

{*Management*} is *Irrigated* or *Rainfed*.

**Date Produced**: January 2021.

**Spatial Metadata**:

Extent: X: −180 to +180

Extent Y: −90 to +90

Extent Z: 12, one layer per month Resolution: 0.083333 decimal degrees (5 arcminutes)

Coordinate reference system: longitude/latitude (WGS84 datum)

Projection in PROJ.4 notation: “+proj = longlat + datum = WGS84”

**Units**: Cropland Area: 1000 ha (10^7^ m^2^) per 5-arcminute grid cell

**No data value:** Oceans, open-water, and Antarctica in the netCDF files have the no-data value of −3.4e + 38.

**Repository**: GeoHub (https://mygeohub.org/)

Note that the annual dataset and the monthly data set are stored in two different repositories. The annual dataset is on Harvard Dataverse and the monthly dataset is on GeoHub. The use of different repositories is based on funding and contract obligations.

## Technical Validation

We compare GAEZ+ 2015 harvested area to FAOSTAT^5^ reported harvested area by crop (Table 3) and by country (Online-only Table 1). It would be useful to also compare harvested area to other global gridded datasets, but at the time of this analysis, there is no other global gridded crop harvested area product for the year 2015. GAEZ+ 2015 crop yields and cropland physical area are compared to other publicly available global gridded datasets for the year 2015, as well as to one dataset for cropland presence/absence at the grid cell level for the year 2010. Validation of any global gridded crop dataset is challenged by universal uncertainties in underlying reported crop statistics and in remote sensing methods. Most, if not all, global gridded crop datasets make use of FAO-reported country level statistics, leading to consistency but not validation when datasets are compared. Here, we report comparisons to other datasets so users familiar with those data are aware of similarities and differences, and we compare spatial aggregates not used in the development of the data, e.g., sub-national boundaries and watershed boundaries, as well as grid cell values where available to illustrate the spatial scale at which these data are consistent or inconsistent.

**Table 3:** Comparison between GAEZ+ 2015 and FAOSTAT global harvested area by crop category.

Crop Name	GAEZ+ 2015 (1,000 ha)	FAOSTAT 2015 (1,000 ha)	Difference (1,000 ha)	Difference (%)*
Wheat	220,620	220,760	−139	−0.06
Maize	190,856	190,639	217	+0.1
Rice	162,633	163,301	−668	−0.4
CropsNES	121,066	121,703	−637	−0.5
Soybean	119,663	120,095	−432	−0.4
Pulses	81,998	76,835	5,162	+7
Vegetables	55,940	56,433	−492	−0.9
Barley	48,331	49,180	−849	−2
Sorghum	43,812	44,279	−468	−1
Rapeseed	34,370	34,519	−149	−0.4
Cotton	32,229	32,214	16	+0.05
Millet	29,933	31,146	−1,213	−4
Sugarcane	26,587	26,771	−183	−0.7
Potato & Sweet potato	26,550	25,896	654	+3
Groundnut	26,279	26,993	−714	−3
Sunflower	25,496	25,659	−163	−0.6
Stimulants	25,266	25,419	−152	−0.6
Cassava	25,231	25,517	−287	−1
Other cereals	25,032	34,450	−9,418	−27
Oil palm fruit	18,357	19,413	−1,056	−6
Yams & other roots	15,992	10,958	5,034	+46
Olives	9,948	10,121	−173	−2
Banana	5,093	10,934	−5,841	−53
Sugarbeet	4,278	4,428	−149	−3
Tobacco	3,725	3,765	−40	−1
**SUM**	**1,379,288**	**1,391,427**	**−12,139**	**−0.9**

*Differences <1% are reported to one significant figure.

### Harvested area comparison

While the goal of this paper is to present year 2015 agricultural data, we first must note any biases apparent in the underlying year 2010 data upon which our product is based, as any bias in the 2010 data will necessarily be carried into the 2015 products. Globally, the sum of all GAEZ v4 2010 harvested areas is 4.2% lower than the FAOSTAT c.2010 total harvested area for all crops, based on all available matching country and crop combinations. The majority of this difference in harvested area is due to the Fruits_&_Nuts category, which is reported in FAOSTAT but not by GAEZ v4 2010. There are also large differences in the Crops_NES and Yams_and_other_roots categories, but in the opposite direction, partially cancelling out the Fruits_&_Nuts discrepancy. The four crops that account for the majority of the world’s production – wheat, maize, rice, and soybeans – all match well, with differences between GAEZ v4 2010 and FAOSTAT c.2010 of <1%. Similarly, the top crop producing countries in the world match well between the two datasets, though notably both Indonesia and Thailand have ~10% less total harvested area in GAEZ v4 2010 than in FAOSTAT c. 2010.

Since no other global gridded harvested area dataset exists at this time, we have only the FAOSTAT country-level and crop total data for 2015 as a basis for comparison. We do not expect the country or global crop aggregates to match exactly because the FAOSTAT data used to generate GAEZ+ 2015 Harvested Area provided only a change in crop harvested area by country; we did not target or calibrate to the FAOSTAT 2015 reported values, and so comparing these two datasets provides some evaluation of the combination of underlying GAEZ 2010 data with the FAOSTAT-based change ratio. Global harvested area by crop from GAEZ+ 2015 and FAOSTAT is shown in Table 3, and total crop harvested area by country from GAEZ+ 2015 and FAOSTAT is shown in Online-only Table 1.

Table 3 presents crops in order of the largest to the smallest global harvested area, according to GAEZ+ 2015. The world’s four staple crops – wheat, maize, rice, and soybeans – all have <1% difference in crop harvested area between FAOSTAT and GAEZ+ 2015. As can be seen in Table 1, the FAOSTAT “CropsNES” category is an aggregate of many crops, which individually have small global harvested areas, but collectively are the third largest harvested area category in the world. Despite challenges in reporting of small crop harvested areas, there is a 0.1% difference in global CropsNES harvested area between FAOSTAT 2015 and GAEZ+ 2015. Notably, GAEZ+ 2015 reports 7% (~5 Mha) more Pulses harvested area than FAOSTAT, a difference that is directly inherited from the 7% difference between GAEZ v4 2010 and FAOSTAT 2010 Pulses harvested area difference. Pulses are the 5^th^ largest harvested area in the world, and an important food crop, particularly in developing countries; this crop warrants more attention from global crop researchers in light of this harvested area difference. Other crop categories with large (>10%) differences are Other cereals, Yams & other roots, and Banana; these crops account for a small proportion of global crop harvested area, but should be evaluated more carefully for local studies of regions where these crops are nutritionally and/or economically important. The GAEZ+ 2015 data set includes 169,182 thousand hectares of fodder crops; this value is not included in Table 3 because the FAO does not report fodder crop area for 2015.

Online-only Table 1 presents harvested area summed for all crops by country, ordered from most to least GAEZ+ 2015 harvested area. The five countries with the most harvested area (India, China, United States of America, Russian Federation, and Brazil) all have less than or equal to 1% difference between GAEZ+ 2015 and FAOSTAT 2015. There are a few notable large countries for which there are larger differences between the datasets; Australia, Germany and France all of more than a 10% difference. There are 31 countries with >0 harvested area reported in FAOSTAT 2015, yet 0 harvested area in GAEZ+ 2015; all these countries have <100,000 ha of FAOSTAT-reported 2015 harvested area, indicating that GAEZ+ 2015 is missing information on many of the smallest agricultural production countries.

### Crop yield comparison

The Global Dataset of Historical Yields (GDHY)^18^ provides a dataset of year 2015 global gridded crop yields for maize, rice, soybean, and wheat. To our knowledge, there are no global gridded datasets of year 2015 yields for the other GAEZ+ 2015 crop categories. Since the GDHY dataset is gridded, it can be used to compare not only yield values (t ha^−1^) but also the spatial distribution of crop yield at the grid cell level.

**Fig. 3:**
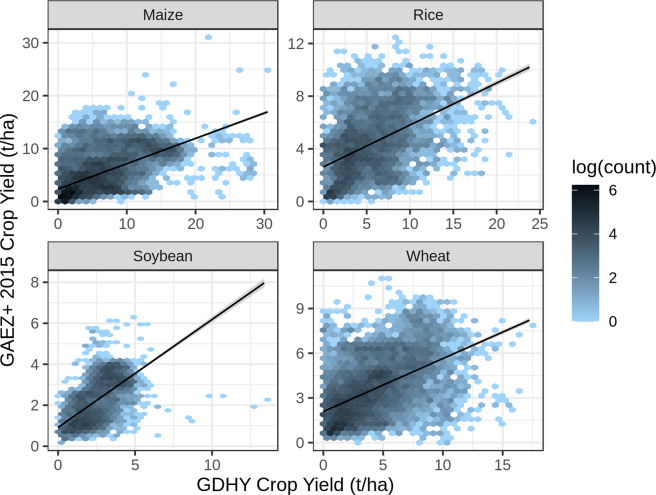
GAEZ+ 2015 grid cell crop yields are consistently lower than Global Dataset of Historical Yields (GDHY)18 grid cell crop yields for all four crops, and with scatter. Hexbin plots show the log of the number of scatter plot points that fall within each hexagon unit on the plot. The linear regression line is shown in black, with 10.1038/s41597-021-01115-2 grey shading around the line showing the 95% confidence interval.

**Fig. 4:**
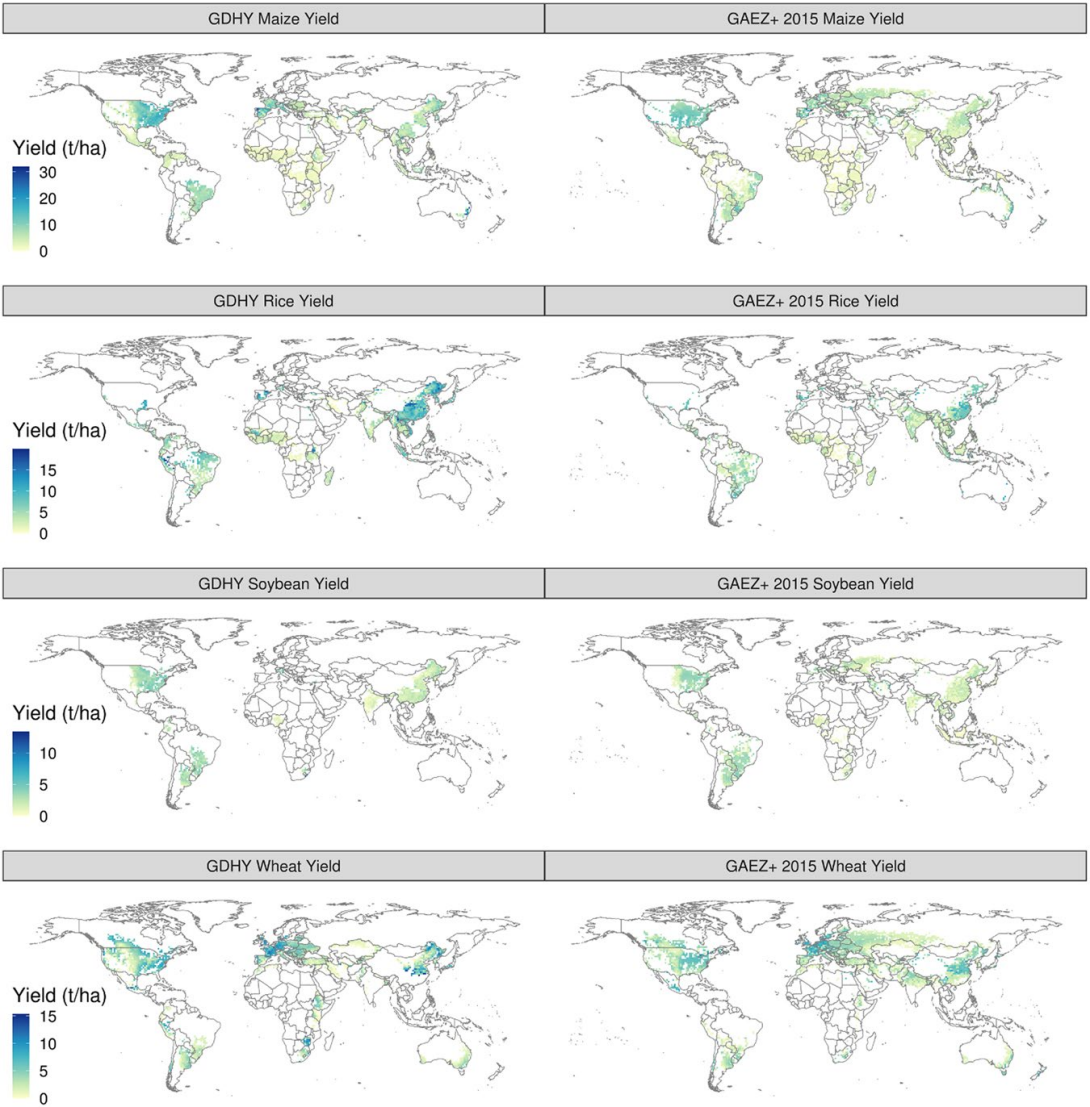
Maps of the Global Dataset of Historical Yields (GDHY)18 and GAEZ+ 2015 grid cell yields (t ha-1) for maize, rice, soybean, and wheat, show agreement on general spatial patterns. Maize, soybean, and wheat show differences in the western parts of Russia and in eastern Europe, with GAEZ+ 2015 reporting a larger spatial extent of these crops.

GAEZ+ 2015 grid cell crop yields are consistently lower than GDHY grid cell crop yields for all four crops (Fig. 3), with grid cell yield linear regression slope values of 0.5, 0.3, 0.9, and 0.4 for maize, rice, soybean, and wheat, respectively. While individual grid cell yield values are consistently lower in GAEZ+ 2015 than GDHY, the former reports a greater spatial extent, with more non-zero grid cell values; this can be seen in the large span of non-zero values along the y-axis of Fig. 3, as well as by comparing maps of the two products (Fig. 4). It is expected that the spatial extent of these two products is different, as the GDHY product uses crop harvested area data from year 2000^19^ as a basis for crop distribution; this difference in distribution, especially the lower extent in GDHY compared to GAEZ+ 2015, explains the difference in grid cell level yields. Yield is a weight per area, so a smaller total area in GDHY necessitates a higher yield per area in order to achieve agreement with the FAO country level yield and production statistics also used by GDHY.

### Cropland physical area comparison

#### Global total

**Fig. 5:**
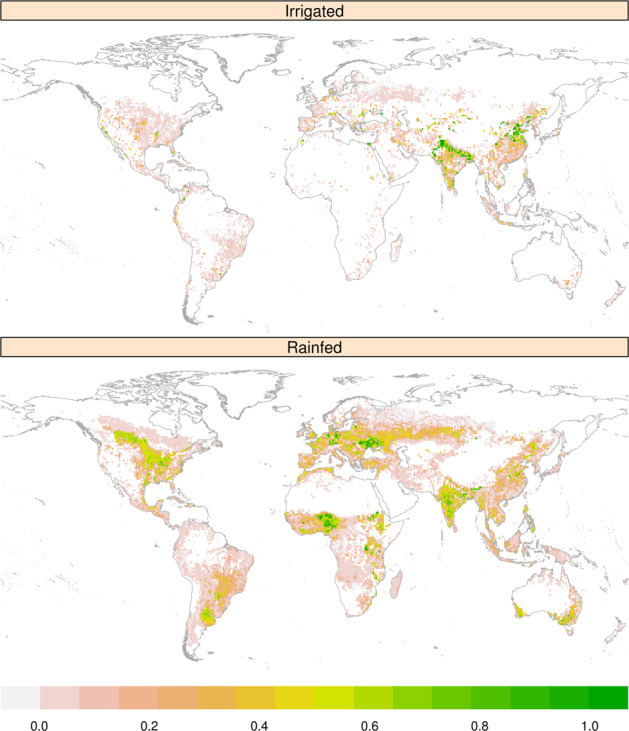
(**a**) GAEZ+ 2015 irrigated cropland, shown as a fraction of each 5-minute grid cell, and (**b**) GAEZ+ 2015 rainfed cropland, shown as a fraction of each 5-minute grid cell.

Annual cropland physical area extent (shown in Fig. 5) can be calculated from the GAEZ+ 2015 monthly cropland physical area data. Here we compare GAEZ+ 2015 cropland extent to FAOSTAT reported cropland area extent^5^. GAEZ+ 2015 minimum cropland extent is calculated by assuming the maximum possible re-use of cropland in multi-cropping systems, effectively using the maximum monthly growing area as the cropland extent. GAEZ+ 2015 maximum cropland extent is calculated by assuming the minimum possible re-use of cropland, effectively taking the minimum of the annual harvested area and the grid cell area as the cropland extent. Globally, we find GAEZ+ 2015 cropland extent is 4–8% lower than FAOSTAT cropland extent (Table 4). This result is consistent with our estimate that GAEZ v4 2010 total harvest area is 4.2% lower than FAOSTAT reported harvested area, and that GAEZ v4 2010 total minimum cropland extent (calculated in the same way as the GAEZ+ 2015 minimum cropland extent) is 10% lower than HYDE 3.2^1^ year 2010 reported global cropland extent.

**Table 4:** Comparison of GAEZ+ 2015 global cropland extent to FAOSTAT year 2015 global cropland extent.

Product	Cropland (ha)	Difference (ha)	Difference (%)
FAOSTAT	1,591,375,400		
GAEZ+ 2015 min	1,467,014,500	−124,360,900	−8
GAEZ+ 2015 max	1,537,518,900	−53,856,500	−4

#### Spatial distribution of cropland physical area

**Fig. 6:**
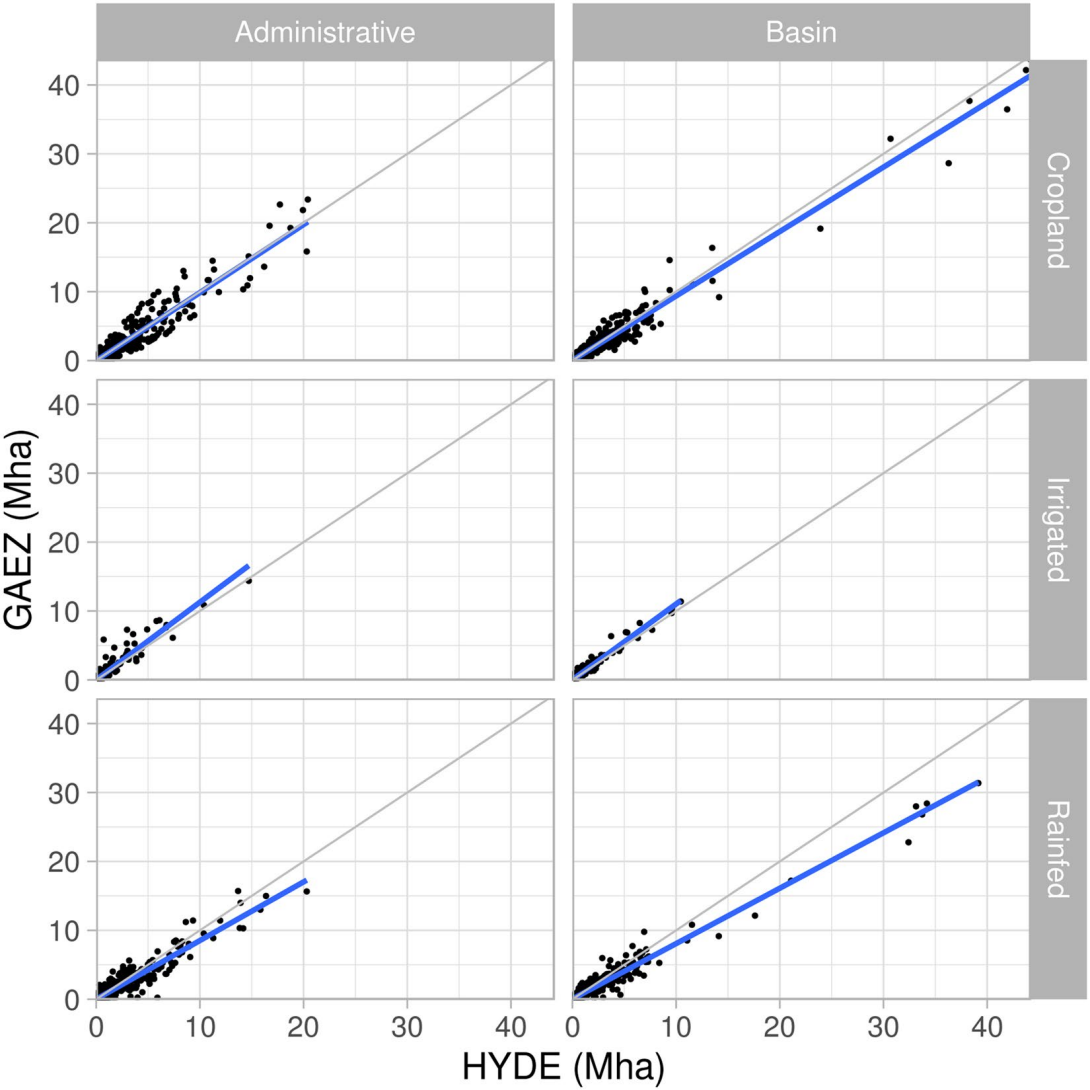
Linear regression of GAEZ+ 2015 (y axis) cropland physical area, irrigated physical area, and rainfed physical area against HYDE 2.31 year 2015 data, aggregated by administrative units and hydrologic basins. Values are in millions of hectares (Mha). The grey line is a 1:1 line, and the blue line is the linear regression model.

**Fig. 7:**
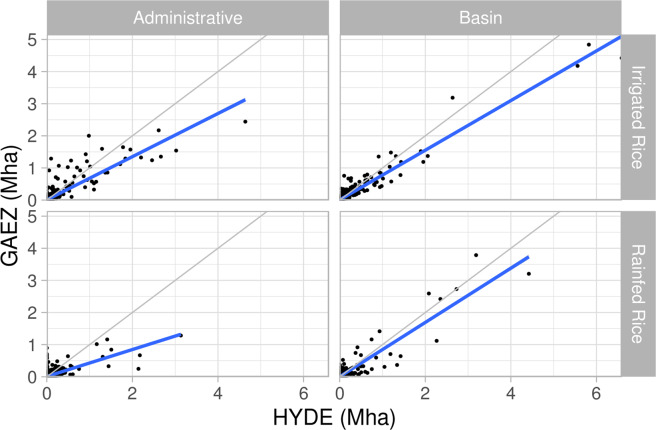
Linear regression of GAEZ+ 2015 (y axis) irrigated rice and rainfed rice physical area against HYDE 2.31 year 2015 data, aggregated by administrative units and hydrologic basins. Values are in millions of hectares (Mha). The grey line is a 1:1 line, and the blue line is the linear regression model.

**Fig. 8:**
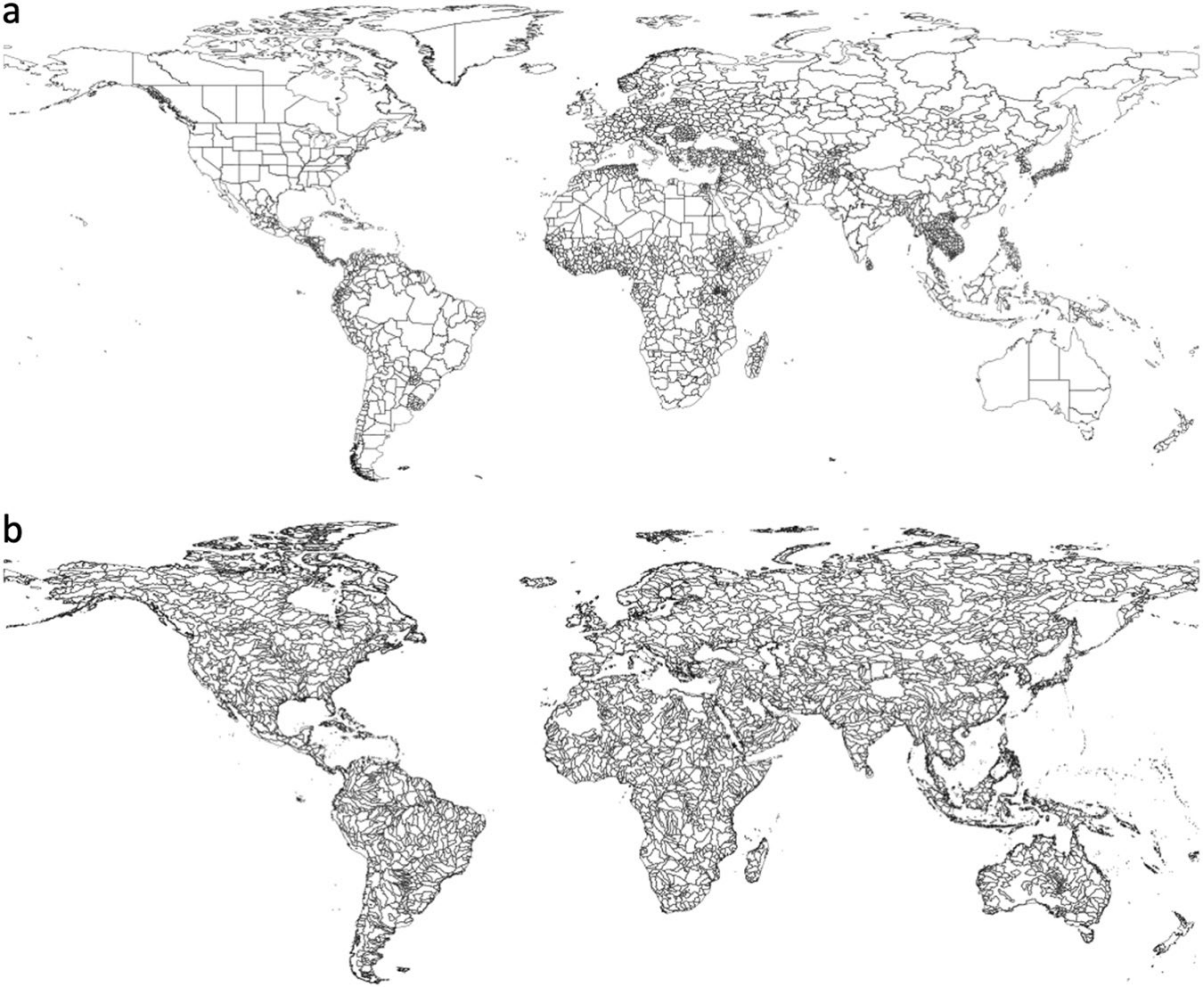
Hydrologic basins (**a**) and administrative units (**b**) used for spatial aggregation. Hydrologic basins defined at 20–200 km^2^ area units (1815 units), derived from the Hydrosheds global 5-arcminute simulated river network^20^, and the administrative units are administrative level 1 data from the FAO GeoNetwork Global Administrative Units Layer (GAUL)^13^ provided for download by^23^.

**Fig. 9:**
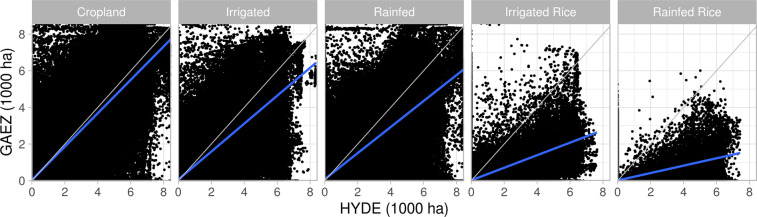
Linear regression of GAEZ+ 2015 (y axis) grid cell cropland physical area, irrigated physical area, rainfed physical area, irrigated rice and rainfed rice physical area against HYDE 2.3^1^ year 2015 grid cell values. Values are in thousands of hectares. The grey line is a 1:1 line, and the blue line is the linear regression model.

To evaluate the accuracy of the GAEZ+ 2015 spatial patterns of cropland physical area, we compare total cropland physical area, irrigated cropland physical area, and rainfed cropland physical area (Fig. 6), as well as irrigated rice physical area and rainfed rice physical area (Fig. 7) to the HYDE 3.2^1^ product. While HYDE 3.2^1^ utilizes country-level information from FAOSTAT within its data generation algorithm, it bases the spatial distribution of cropland on remote sensing data, which provides an independent source of sub-national crop data. Validation was done at two spatial aggregates: (1) hydrologic basins defined at 20,000–200,000 km^2^ area units (1,815 unique units), derived from the Hydrosheds global 5-arcminute simulated river network^20^ and (2) administrative boundaries at the sub-national level (administrative level 1 from the FAO GeoNetwork Global Administrative Units Layer (GAUL)^13^ provided for download by^18^ (See Fig. 8 for maps of the spatial units). This provides validation metrics at geophysical and socio-political scales relevant to the modelling communities that would use this dataset. We also compare grid cell values of physical areas for all categories available from HYDE (Fig. 9, Table 5).

**Table 5:** Linear regression results comparing the spatial distribution of GAEZ+ 2015 cropland physical area to HYDE 2.3^1^ cropland by administrative unit, hydrologic basin aggregation, and individual grid cells.

	Administrative units			Hydrologic Basins			Grid Cells		
	r2	slope	RMSE (Mha)	r2	slope	RMSE (Mha)	r2	slope	RMSE (1,000 ha)
Total cropland	0.92	0.99	0.492	0.96	0.94	0.502	0.79	0.91	0.66
Irrigated physical area	0.9	1.13	0.149	0.96	1.1	0.223	0.59	0.76	0.46
Rainfed physical area	0.91	0.85	0.364	0.96	0.8	0.373	0.69	0.71	0.60
Irrigated Rice physical area	0.77	0.67	0.058	0.94	0.77	0.072	0.36	0.34	0.18
Rainfed Rice physical area	0.48	0.42	0.068	0.85	0.84	0.053	0.19	0.20	0.13

Slope values <1 indicate GAEZ+ 2015 under-reports physical area compared to HYDE 2.3; >1 indicates over-reporting. Note that the RMSE units are Mha for the spatial aggregates, and 1,000 ha for grid cells.

Linear regression results (Table 5 and Figs. 6, 7, and 9) show that when aggregated spatially, GAEZ+ 2015 minimum cropland, irrigated, and rainfed physical area match well with HYDE 3.2 spatial distributions (r^2^ ≥ 0.9 for all three), with results significant at the p < 0.001 level. With the exception of irrigated land, the slope of the regression of GAEZ+ 2015 as a function of HYDE 2.3 is slightly less than 1.0, indicating a consistent small underestimation of rainfed land, and small overestimation of irrigated land compared to HYDE 3.2. However, the strength of the linear regression shows that the spatial distributions are similar. Rice irrigated and rainfed physical areas match less well, with r^2^ values of only 0.77 and 0.48 for irrigated and rainfed, respectively, at the administrative unit level (values are higher for the basin aggregations). Differences in rice physical area are not surprising, especially for rainfed rice, given the known challenges of mapping widely distributed and diverse rice cropping systems, e.g.^21,22^.

While the linear regression shows agreement in aggregate, the grid cell comparison (Fig. 9) illustrates large scatter, especially in cropland physical area. This large scatter is consistent with the differences in underlying methods used to identify crop area presence/absence in the two datasets. We expect GAEZ+ 2015 to have 0 or lower values where HYDE reports non-zero values due to the use of the GAEZ v4 2010 crop map as a basis for the GAEZ+ 2015 data product.

### Grid cell crop presence/absence comparison for year 2010

Lastly, we compare the GAEZ v4 data that underlies GAEZ+ 2015 to the MapSPAM^10^ crop data product. While this paper presents the new GAEZ+ 2015 dataset, note that the methods used to produce this gridded data restrict a given crop’s presence (harvested area, yield, and physical area) to the grid cells in which that crop was present in the GAEZ v4 data product. Therefore, we find it informative to compare the GAEZ v4 crop-specific presence/absence to another data set that provides such information for the same year (2010); this will provide a view of how the underlying crop map data compares to other published data, and display the constraints placed on the GAEZ+ 2015 crop presence/absence in grid cells.

**Fig. 10:**
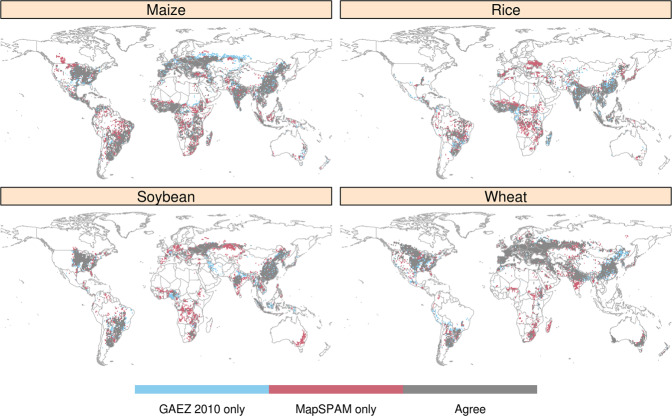
Agreement (grey), and disagreement (blue and red) between GAEZ v4 and MapSPAM10 year 2010 presence of maize, rice, soybean, and wheat. Grey indicates the crop is present in both datasets; blue shows the crop is present in GAEZ v4 but absent in MapSPAM, and red shows the crop is present in MapSPAM but absent in GAEZ v4.

With a few notable exceptions, grid cell presence/absence of the world’s four main crops – maize, rice, soybean, and wheat – mostly agree between GAEZ v4 and MapSPAM (Fig. 10). For all four crops, MapSPAM has presence in more grid cells than GAEZ v4 across Sub-Saharan Africa. There are also crop-specific inconsistencies across western Canada (Maize), Europe (Rice), Australia and Russia (Soybean), and India (Wheat).

## Usage Notes

There are several known issues and limitations that users should be aware of. These are described in the following paragraphs.

### Annual harvested area versus monthly cropland area discrepancies

As noted in the Methods section above, there is a small disparity between the annual harvested area and the monthly cropland area for some crops in the GAEZ+ 2015 product; the maximum difference in any month is 0.05% of global cropland.

### Irrigated cassava files

The annual product includes a file for irrigated cassava harvested area. All grid cell areas for this crop are 0; this follows from zero irrigated cassava area in 2010 in GAEZ v4 data; therefore we did not include irrigated cassava in the monthly product.

### Limitation of the country-level statistics approach

This product will become obsolete when a product becomes available that updates global data using the GAEZ or a similar methodology to account for sub-national shifts in the spatial distribution of cropland due to a range of a potential factors, such as land protection, land degradation, urban expansion, infrastructure development such as roads or reservoirs and canals, or climate change. The use of country-level statics in the methods presented here cannot capture these changes in spatial distribution; therefore, this product should be seen as a temporary tool to be used while researchers are waiting for updated products that can capture those changes.

### Lack of new information on irrigation

There was insufficient post-2010 irrigation data available from FAO to update the distribution of irrigation activity among crop types and land areas, so the product uses for 2015 the same gridded rainfed-to-irrigated crop harvest area and crop production ratios as reported in GAEZ v4 for 2010.

### Lack of fallow land information

Fallow land is not included as a category in this dataset. This is partly due to a compromise made in the development of the dataset: because the grid cells with presence of a given crop are restricted to the same grid cells from GAEZ v4, we allowed the total cropland extent within a grid cell to become large in order to best match national level statistics. This accommodates cases where the crop expanded to other grid cells, yet leaves little room for fallow land.

### Lagging data usage

It is a challenge to keep global cropland products current – the global agricultural system is constantly in flux, yet global data products take time to develop, so their input data is necessarily from earlier years. Some challenging features to quantify (e.g., crop calendars, irrigation) are only re-constructed at the global scale very infrequently. For example, GAEZ+ 2015 monthly crop data relies on the MIRCA2000 data^4^ to characterize sub-annual cropping activity; MIRCA2000 was published a decade ago and represents the situation c.2000, a decade and a half prior to the 2015 target year of this new data set.

### Other uncertainties

Any global agricultural data set will contain errors. These could result from imperfect statistical reporting of underlying data, simplifications needed to successfully harmonize disparate data sets (e.g., cropland data and irrigation data), and inherent uncertainties in remote sensing characterization of global-scale land use.

This product necessarily carries forward any errors in the 2010 base product of GAEZ v4.

Finally, we note that due to the limitations – especially the country-level statistics approach – model results generated using this dataset should be interpreted with care at the level of grid cells are small aggregates. While gridded input data is required for many global models, we recommend interpreting results at aggregate spatial levels such as the administrative units or watersheds presented in Fig. 8. Grid cell level data such as MIRCA2000, HYDE 2.3, and MapSPAM are routinely used in global hydrologic models attempting to simulate years not represented by those datasets; here, the GAEZ+ 2015 update to the GAEZ v4 product aims to incorporate updated crop data into an existing gridded product to reduce the bias resulting from using outdated data. This method generates its own, unique set of biases due to the limitations described above.

## Data Availability

Code used in this paper is available here: https://github.com/wsag/GAEZ-_2015_code. This repository includes the scripts that: a) Convert annual harvested area to monthly crop physical area b) Aggregate the Hydrosheds 5-minute river network into 20–200 km^2^ watersheds c) Compares GAEZ+ 2015 harvested area with FAOSTAT harvested area d) Compares GAEZ+ 2015 yield data to GDHY yield data e) Compares GAEZ+ 2015 cropland physical area data to HYDE 2.3 cropland physical area data

## Online-only Table

**Online-only Table 1:** Comparison between GAEZ+ 2015 and FAOSTAT country-level harvested area.

ADM0 Name	GAEZ+ 2015 (1000 ha)	FAOSTAT 2015 (1000 ha)	Difference (1000 ha)	Difference (%)
India	197,327	198,400	−1,073	−1
China	182,526	181,049	1,477	1
United States of America	102,979	103,127	−148	0
Russian Federation	76,133	76,437	−304	0
Brazil	59,769	59,933	−165	0
Nigeria	50,779	52,024	−1,245	−2
Australia	38,600	43,055	−4,456	−10
Argentina	35,066	35,027	39	0
Indonesia	29,022	29,319	−297	−1
Canada	25,091	25,191	−100	0
Ukraine	22,826	23,243	−417	−2
Pakistan	22,195	21,384	811	4
Germany	18,569	16,395	2,175	13
Kazakhstan	18,134	17,970	165	1
Turkey	17,531	17,540	−9	0
France	17,301	19,794	−2,493	−13
Sudan	17,272	17,532	−260	−1
Mexico	16,958	17,103	−146	−1
Thailand	16,899	17,142	−243	−1
Myanmar	16,560	16,659	−99	−1
United Republic of Tanzania	15,817	15,489	328	2
Niger	15,439	15,696	−257	−2
Ethiopia	14,248	14,063	185	1
Bangladesh	14,159	14,513	−355	−2
Philippines	14,091	14,205	−114	−1
Viet Nam	12,851	12,815	36	0
Spain	12,602	12,735	−133	−1
Iran (Islamic Republic of)	11,265	11,813	−547	−5
Poland	10,461	10,901	−439	−4
Democratic Republic of the Congo	9,891	9,824	67	1
Cote d’Ivoire	8,824	8,855	−31	0
Romania	8,254	6,711	1,542	23
Italy	7,901	7,893	8	0
Paraguay	7,053	6,842	211	3
Uganda	6,928	7,038	−110	−2
Morocco	6,850	6,833	16	0
Mali	6,778	6,741	38	1
South Africa	6,757	7,058	−300	−4
Cameroon	6,613	6,687	−74	−1
Egypt	5,839	5,867	−28	0
Ghana	5,800	5,792	8	0
Burkina Faso	5,576	5,563	13	0
U.K. of Great Britain and Northern Ireland	5,449	5,555	−106	−2
Kenya	5,407	5,391	16	0
Belarus	5,236	7,043	−1,807	−26
Mozambique	5,050	5,158	−109	−2
Malaysia	5,036	5,010	26	1
Algeria	4,485	5,039	−553	−11
Angola	4,398	4,441	−43	−1
Nepal	4,319	4,362	−44	−1
Uzbekistan	4,246	4,245	1	0
Guinea	4,201	4,424	−224	−5
Hungary	4,162	4,470	−308	−7
Syrian Arab Republic	3,954	3,941	13	0
Malawi	3,882	5,019	−1,138	−23
Japan	3,737	3,776	−39	−1
Colombia	3,714	4,280	−566	−13
Chad	3,656	3,681	−25	−1
Bolivia	3,525	3,558	−33	−1
Cambodia	3,453	3,482	−29	−1
Tunisia	3,382	3,384	−2	0
Afghanistan	3,359	3,366	−7	0
Peru	3,200	3,285	−84	−3
Benin	3,091	3,092	−1	0
Bulgaria	2,928	2,670	258	10
Madagascar	2,896	2,909	−14	0
Serbia	2,752	2,721	31	1
Turkmenistan	2,693	2,737	−44	−2
Greece	2,555	2,564	−9	0
Senegal	2,501	2,503	−2	0
Dem People’s Rep of Korea	2,462	2,607	−146	−6
Lithuania	2,439	2,011	428	21
Iraq	2,405	2,314	91	4
Denmark	2,294	2,445	−151	−6
Czech Republic	2,290	2,247	44	2
Ecuador	2,261	2,190	72	3
Sweden	2,253	2,238	15	1
Guatemala	2,195	2,233	−39	−2
Uruguay	2,175	2,320	−145	−6
Zimbabwe	2,123	1,889	234	12
Zambia	2,030	2,019	11	1
Togo	1,966	2,259	−293	−13
Sri Lanka	1,788	1,745	44	2
Rwanda	1,759	1,740	18	1
Latvia	1,684	1,664	20	1
South Sudan	1,674	1,738	−64	−4
Portugal	1,614	1,583	31	2
Azerbaijan	1,507	1,602	−95	−6
Lao People’s Democratic Republic	1,497	1,554	−57	−4
Finland	1,446	1,530	−85	−6
Moldova Republic of	1,438	1,479	−41	−3
Chile	1,432	1,345	88	7
Sierra Leone	1,335	1,358	−23	−2
Haiti	1,238	1,260	−22	−2
Republic of Korea	1,217	1,064	153	14
Venezuela	1,205	1,234	−29	−2
Cuba	1,146	1,159	−13	−1
Netherlands	1,119	1,101	18	2
Austria	1,070	1,069	1	0
Burundi	1,070	1,097	−27	−2
Slovakia	1,050	1,089	−39	−4
Kyrgyzstan	1,030	1,024	6	1
Yemen	963	962	0	0
Honduras	924	952	−28	−3
Papua New Guinea	905	923	−19	−2
Nicaragua	894	900	−6	−1
Belgium	845	915	−70	−8
Tajikistan	836	833	4	0
Central African Republic	829	837	−7	−1
Libya	777	799	−22	−3
Estonia	771	785	−14	−2
Dominican Republic	719	694	25	4
Croatia	629	618	11	2
El Salvador	618	636	−18	−3
Norway	594	600	−6	−1
Taiwan	589	589	1	0
Saudi Arabia	570	636	−66	−10
Bosnia and Herzegovina	540	532	8	2
Somalia	535	522	13	2
Eritrea	522	516	6	1
Liberia	497	542	−45	−8
Ireland	476	445	31	7
Albania	463	470	−7	−1
Costa Rica	382	365	16	4
New Zealand	381	390	−10	−2
Guinea-Bissau	377	359	18	5
Namibia	372	368	4	1
Armenia	363	380	−16	−4
Switzerland	329	331	−3	−1
Mongolia	322	330	−8	−3
Georgia	320	292	27	9
Congo	298	314	−16	−5
Mauritania	291	291	1	0
Gambia	288	287	1	1
The former Yugoslav Republic of Macedonia	282	269	13	5
Israel	269	278	−9	−3
Panama	260	311	−50	−16
Gabon	248	247	1	0
Guyana	247	252	−5	−2
Jordan	237	199	38	19
Lebanon	230	244	−14	−6
Slovenia	184	193	−8	−4
Timor-Leste	180	185	−5	−3
Swaziland	148	145	3	2
Botswana	147	139	8	6
Jamaica	145	138	7	5
Lesotho	145	146	−2	−1
Montenegro	125	100	24	24
Bhutan	108	109	0	0
Vanuatu	100	99	0	0
Fiji	95	0	95	NA
West Bank	93	14	79	569
Solomon Islands	92	107	−15	−14
Belize	91	101	−10	−10
Equatorial Guinea	91	91	0	0
United Arab Emirates	73	73	0	0
Cyprus	66	63	4	6
Luxembourg	57	58	−1	−2
Suriname	55	55	0	−1
Oman	41	35	6	17
Jammu and Kashmir	39	0	39	NA
Arunachal Pradesh	35	0	35	NA
Puerto Rico	34	20	15	75
Guadeloupe	19	21	−2	−9
Kuwait	16	17	−1	−6
New Caledonia	15	10	4	43
Malta	14	15	−1	−10
Abyei	14	0	14	NA
French Guiana	13	0	13	NA
Gaza Strip	13	14	−1	−9
Brunei Darussalam	12	13	0	−1
Bahamas	9	9	1	7
Qatar	5	5	1	13
Madeira Islands	4	5	−1	−23
Bahrain	3	3	0	6
Antigua and Barbuda	2	2	0	−19
Hong Kong	2	0	2	NA
Jersey	1	0	1	NA
Guernsey	0.33	0.00	0.33	NA
San Marino	0.29	0.00	0.29	NA
Ilemi triangle	0.28	0.00	0.28	NA
Liechtenstein	0.17	1.39	−1.22	−87.61
Singapore	0.15	0.00	0.15	NA
Andorra	0.09	0.00	0.09	NA
China/India	0.04	0.00	0.04	NA
Macau	0.03	0.13	−0.10	−75.58
Djibouti	0.02	12.30	−12.28	−99.82
Monaco	0.01	0.00	0.01	NA
Western Sahara	0.01	3.55	−3.54	−99.79
Holy See	0.00	0.00	0.00	
Aksai Chin	0.00	0.00	0.00	
American Samoa	0.00	6.24	−6.24	−100
Anguilla	0.00	0.00	0.00	
Aruba	0.00	0.00	0.00	
Ashmore and Cartier Islands	0.00	0.00	0.00	
Azores Islands	0.00	0.00	0.00	
Baker Island	0.00	0.00	0.00	
Barbados	0.00	6.03	−6.03	−100
Bassas da India	0.00	0.00	0.00	
Bermuda	0.00	0.34	−0.34	−100
Bird Island	0.00	0.00	0.00	
Bouvet Island	0.00	0.00	0.00	
British Indian Ocean Territory	0.00	0.00	0.00	
British Virgin Islands	0.00	0.07	−0.07	−100
Cape Verde	0.00	71.70	−71.70	−100
Cayman Islands	0.00	0.31	−0.31	−100
Christmas Island	0.00	0.00	0.00	
Clipperton Island	0.00	0.00	0.00	
Cocos (Keeling) Islands	0.00	0.00	0.00	
Comoros	0.00	117.32	−117.32	−100
Cook Islands	0.00	1.68	−1.68	−100
Dominica	0.00	20.69	−20.69	−100
Europa Island	0.00	0.00	0.00	
Falkland Islands (Malvinas)	0.00	0.00	0.00	
Faroe Islands	0.00	0.11	−0.11	−100
French Polynesia	0.00	25.41	−25.41	−100
French Southern and Antarctic Territories	0.00	0.00	0.00	
Gibraltar	0.00	0.00	0.00	
Glorioso Island	0.00	0.00	0.00	
Greenland	0.00	0.00	0.00	
Grenada	0.00	11.79	−11.79	−100
Guam	0.00	8.29	−8.29	−100
Hala’ib triangle	0.00	0.00	0.00	
Heard Island and McDonald Islands	0.00	0.00	0.00	
Howland Island	0.00	0.00	0.00	
Iceland	0.00	2.44	−2.44	−100
Isle of Man	0.00	0.00	0.00	
Jarvis Island	0.00	0.00	0.00	
Johnston Atoll	0.00	0.00	0.00	
Juan de Nova Island	0.00	0.00	0.00	
Kingman Reef	0.00	0.00	0.00	
Kiribati	0.00	24.74	−24.74	−100
Kuril islands	0.00	0.00	0.00	
Maldives	0.00	3.16	−3.16	−100
Marshall Islands	0.00	7.60	−7.60	−100
Martinique	0.00	16.44	−16.44	−100
Ma’tan al-Sarra	0.00	0.00	0.00	
Mauritius	0.00	61.87	−61.87	−100
Mayotte	0.00	0.00	0.00	
Micronesia (Federated States of)	0.00	21.02	−21.02	−100
Midway Island	0.00	0.00	0.00	
Montserrat	0.00	0.46	−0.46	−100
Nauru	0.00	0.46	−0.46	−100
Navassa Island	0.00	0.00	0.00	
Netherlands Antilles	0.00	0.00	0.00	
Niue	0.00	5.32	−5.32	−100
Norfolk Island	0.00	0.00	0.00	
Northern Mariana Islands	0.00	0.00	0.00	
Palau	0.00	0.00	0.00	
Palmyra Atoll	0.00	0.00	0.00	
Paracel Islands	0.00	0.00	0.00	
Pitcairn	0.00	0.00	0.00	
R√©union	0.00	48.41	−48.41	−100
Saint Helena	0.00	0.00	0.00	
Saint Kitts and Nevis	0.00	1.45	−1.45	−100
Saint Lucia	0.00	7.15	−7.15	−100
Saint Pierre et Miquelon	0.00	0.01	−0.01	−100
Saint Vincent and the Grenadines	0.00	14.64	−14.64	−100
Samoa	0.00	47.08	−47.08	−100
Sao Tome and Principe	0.00	43.03	−43.03	−100
Scarborough Reef	0.00	0.00	0.00	
Senkaku Islands	0.00	0.00	0.00	
Seychelles	0.00	2.13	−2.13	−100
South Georgia and the South Sandwich Islands	0.00	0.00	0.00	
Spratly Islands	0.00	0.00	0.00	
Svalbard and Jan Mayen Islands	0.00	0.00	0.00	
Tokelau	0.00	0.62	−0.62	−100
Tonga	0.00	24.31	−24.31	−100
Trinidad and Tobago	0.00	30.05	−30.05	−100
Tromelin Island	0.00	0.00	0.00	
Turks and Caicos islands	0.00	0.00	0.00	
Tuvalu	0.00	1.85	−1.85	−100
United States Virgin Islands	0.00	0.00	0.00	
Wake Island	0.00	0.00	0.00	
Wallis and Futuna	0.00	7.05	−7.05	−100
**SUM:**	**1,379,288**	**1,391,427**	**−12,139**	**−1%**

Countries with ≥1,000 ha have harvested area values reported to 1 (1,000) ha; countries with <1,000 ha total harvested are reported to 10 ha (or 0.01 (1,000) ha units).

*Differences <1% are reported to one significant figure. Countries with 0 harvested area reported either by FAOSTAT or GAEZ+ 2015 have a % difference value of NA. Countries with 0 harvested area reported both by FAOSTAT and GAEZ+ 2015 (agreement) have no % difference reported, as the two datasets have no difference but the formula for calculating percent different is undefined.^10^

## References

[1] Klein Goldewijk K, Beusen A, Doelman J, Stehfest E (2017). Anthropogenic land use estimates for the Holocene – HYDE 3.2. Earth Syst. Sci. Data.

[2] Ramankutty, N., Evan, A. T., Monfreda, C. & Foley, J. A. Farming the planet: 1. Geographic distribution of global agricultural lands in the year 2000: GLOBAL AGRICULTURAL LANDS IN 2000. *Glob. Biogeochem. Cycles* **22** (2008).

[3] Geddes, J. A., Martin, R. V., Brauer, M., Boys, B. L. & vanDonkelaar, A. Global Agricultural Lands: Croplands, 2000, 10.7927/H4C8276G (2010).

[4] Portmann, F. T., Siebert, S. & Döll, P. MIRCA2000-Global monthly irrigated and rainfed crop areas around the year 2000: A new high-resolution data set for agricultural and hydrological modeling: MONTHLY IRRIGATED AND RAINFED CROP AREAS. *Glob. Biogeochem. Cycles* **24** (2010).

[5] Food and Agriculture Organization of the United Nations. FAOSTAT Statistical Database. [Rome]:FAO, 1997. Accessed April 2020.

[6] Fischer, G. *et al*. Global Agro-ecological Zones (GAEZ v4)- Model Documentation. FAO & IIASA, Luxemburg, Austria; Rome, Italy, 10.4060/cb4744en (2021).

[7] FAO and IIASA. Global Agro Ecological Zones version 4 (GAEZ v4). Accessed June 2021. http://www.fao.org/gaez/ (2021).

[8] Fischer, G., T. Ermolieva, Y. Ermoliev, H. van Velthuizen. Spatial recovering of agricultural values from aggregate information: Sequential downscaling methods. *Intern. J. Knowledge and Systems Sciences*, **3**(1) (2006).

[9] Thenkabail, P. *et al* NASA Making Earth System Data Records for Use in Research Environments (MEaSUREs) Global Food Security Support Analysis Data (GFSAD) Crop Dominance 2010 Global 1 km V001. *NASA EOSDIS Land Processes DAAC*, 10.5067/MEaSUREs/GFSAD/GFSAD1KCD.001 (2016).

[10] International Food Policy Research Institute. Global Spatially-Disaggregated Crop Production Statistics Data for 2010 Version 2.0. *Harvard Dataverse*, 10.7910/DVN/PRFF8V (2019).

[11] Baldos ULC, Haqiqi I, Hertel TW, Horridge M, Liu J (2020). SIMPLE-G: A multiscale framework for integration of economic and biophysical determinants of sustainability. Environ. Model. Softw..

[12] Calvin K (2019). GCAM v5.1: representing the linkages between energy, water, land, climate, and economic systems. Geosci. Model Dev..

[13] Food and Agricultural Organization of the United Nations, FAO GeoNetwork. Global Administrative Unit Layer. *FAO Map Catalog* https://data.apps.fao.org/map/catalog/srv/eng/catalog.search/metadata/9c35ba10-5649-41c8-bdfc-eb78e9e65654 (2012).

[14] Frolking S, Wisser D, Grogan D, Proussevitch A, Glidden S (2020). Harvard Dataverse.

[15] Frolking S, Wisser D, Grogan D, Proussevitch A, Glidden S (2020). Harvard Dataverse.

[16] Frolking S, Wisser D, Grogan D, Proussevitch A, Glidden S (2020). Harvard Dataverse.

[17] Grogan D, Prusevich A, Frolking S, Wisser D, Glidden S (2021). MyGeoHub.

[18] Iizumi T, Sakai T (2020). The global dataset of historical yields for major crops 1981–2016. Sci Data.

[19] Monfreda C, Ramankutty N, Foley JA (2008). Farming the planet: 2. Geographic distribution of crop areas, yields, physiological types, and net primary production in the year 2000, *Global Biogeochem*. Cycles.

[20] Lehner B, Verdin K, Jarvis A (2008). New Global Hydrography Derived From Spaceborne Elevation Data. Eos Trans. Am. Geophys. Union.

[21] Dong J, Xiao X (2016). Evolution of regional to global paddy rice mapping methods: A review. ISPRS Journal of Photogrammetry and Remote Sensing.

[22] Gumma MK, Nelson A, Thenkabail PS, Singh AN (2011). Mapping rice areas of South Asia using MODIS multitemporal data. Journal of applied remote sensing.

[23] Urbano, F. Global administrative boundaries. European Commission, Joint Research Centre (JRC). *Joint Research Centre Data Catalogue* http://data.europa.eu/89h/jrc-10112-10004 (2018).

